# The Malmö Offspring Study (MOS): design, methods and first results

**DOI:** 10.1007/s10654-020-00695-4

**Published:** 2020-11-21

**Authors:** Louise Brunkwall, Daniel Jönsson, Ulrika Ericson, Sophie Hellstrand, Cecilia Kennbäck, Gerd Östling, Amra Jujic, Olle Melander, Gunnar Engström, Jan Nilsson, Bodil Ohlsson, Björn Klinge, Marju Orho-Melander, Margaretha Persson, Peter M. Nilsson

**Affiliations:** 1 Department of Clinical Sciences, Lund University, Malmö, Sweden; 2 Faculty of Dentistry, Malmö University, Malmö, Sweden; 3 Department of Internal Medicine, Skane University Hospital, Jan Waldenströms gata 15, 5th floor, 20502 Malmö, Sweden; 4 Department of Cardiology, Skane University Hospital, Malmö, Sweden

**Keywords:** Cardio metabolic, Diet, Family, Microbiota, Offspring, Vascular

## Abstract

As cardio metabolic disease manifestations tend to cluster in families there is a need to better understand the underlying mechanisms in order to further develop preventive strategies. In fact, genetic markers used in genetic risk scores, important as they are, will not be able alone to explain these family clusters. Therefore, the search goes on for the so called *missing heritability* to better explain these associations. Shared lifestyle and social conditions in families, but also early life influences may be of importance. Gene-environmental interactions should be explored. In recent years interest has grown for the role of diet-microbiota associations, as microbiota patterns may be shared by family members. In the Malmö Offspring Study that started in 2013, we have so far been able to examine about 4700 subjects (18–71 years) representing children and grandchildren of index subjects from the first generation, examined in the Malmö Diet Cancer Study during 1991 to 1996. This will provide rich data and opportunities to analyse family traits of chronic disease across three generations. We will provide extensive genotyping and phenotyping including cardiovascular and respiratory function, as well as markers of glucose metabolism. In addition, also cognitive function will be assessed. A 4-day online dietary recall will be conducted and gut as well as oral microbiota analysed. The ambition is to provide one of the first large-scale European family studies with individual data across three generations, which could deepen our knowledge about the role of family traits for chronic disease and its underlying mechanisms.

**Electronic supplementary material:**

The online version of this article (10.1007/s10654-020-00695-4) contains supplementary material, which is available to authorized users.

## Introduction

The Global Burden of Disease (GBD) project reports non-communicable cardio metabolic conditions and diseases, such as obesity, type 2 diabetes (T2D), and cardiovascular disease (CVD), together with cancer, to continue to increase world-wide, providing enormous demands on future health-care systems [1]. These conditions have previously been described as diseases of wealth, but today their prevalence is increasing more rapidly than wealth around the world. Therefore, among the greatest challenges of medical sciences is to find novel and more effective ways to prevent and treat these diseases. For this purpose, it is important that large population- and family-based cohorts of individuals exposed to present-day environmental factors are collected and investigated.

A positive family history of early onset disease is a strong risk factor for cardio metabolic diseases [2]. Familial influence acts through a combination of genetic and epigenetic factors as well as shared family background, including many environmental influences such as diet, other lifestyle habits, oral-health and psychosocial- and socioeconomic factors. In order to design better strategies for prevention and treatment of chronic diseases and their complications, a deeper understanding of relevant family traits is needed. During the last decade, genome-wide association studies (GWAS) and Mendelian randomization studies have importantly contributed to the identification of novel disease mechanisms, causal effects and development of new drug targets [3, 4]. Moreover, studies of the gut microbiota have linked the faecal bacterial composition, also a partially heritable trait [5], to numerous chronic diseases including obesity, T2D [6] and CVD. Increasing evidence supports the view that gut microbiota may operate as a key mediator for adverse lifestyle risk factors such as unhealthy diet [7]. Further, oral health and in particular the severity of periodontitis has been associated with CVD and T2D through chronic low-grade systematic inflammation [8–10], and studies of oral microbiota open new possibilities to clarify this link. Nevertheless, despite these important contributions, the heritability of most chronic diseases and traits has remained largely unexplained and cannot solely be attributed to the thus far identified genetic risk factors, as exemplified by T2D [11]. This has been called the *missing heritability* challenge, and needs to be further explored, preferably in population-based family studies.

Internationally, only few studies have investigated family health traits across generations, one example being the well-known Framingham Offspring Study (FOS), where the children and grandchildren of index individuals have been invited for repeated screening exams [12, 13]. In most observational studies, the family history is evaluated based on self-reported data from questionnaires. In Sweden, the unique national registers can be utilized to evaluate the more detailed family burden of disease and to longitudinally follow-up the morbidity and mortality patterns of population- and family studies, for example the family burden of CVD in the Malmö Preventive Project (MPP) [14].

The Malmö Offspring Study (MOS) was initiated in 2013 and is an ongoing large-scale, family-based cohort study with register-based follow-up by use of the personal identification system in Sweden. MOS participants represent adult children and grandchildren of participants from an earlier cohort study launched in the 1990s, the Malmö Diet and Cancer Study (MDCS). With a mean follow-up of more than 20 years, MDCS provides exceptionally detailed information about the medical aspects of family history of MOS participants as well as lifestyle and dietary information, the latter with exceptional high quality [15, 16].

## Aim

The *general aim *of MOS is to map risk factors of importance for family traits of chronic diseases including genetic-, epigenetic- and circulating biomarkers, gut and oral microbiota, vascular imaging, family history, medical history, cognitive function, diet, other lifestyle factors and social aspects. Risk factor levels in parents will be linked to adverse risk factors levels, vascular changes, cognitive function, as well as oral health, and prognosis in their offspring. In addition, the national Multi-Generation Register (MGR) will be utilized to evaluate the family burden of chronic diseases, based on data not only in parents and offspring but also in siblings and other close relatives. Finally, MOS is aiming to include over 5000 participants, and represents in itself a new cohort for future follow-up studies.

## Study design

### Source population: index generation 1

The study participants of MOS consists of invited adult children and grandchildren to participants in the Malmö Diet and Cancer Study—Cardiovascular Cohort (MDCS-CC, n = 6103), which is a random, deeply phenotyped subpopulation of the Malmö Diet and Cancer Study (MDCS) [17]. MDCS is a large population-based cohort originally designed to investigate the relationship between a diet rich in fat and low in fibre, and several different cancer forms. Baseline examinations were performed between 1991 and 1996 when 28,098 then middle-aged individuals completed the examinations. MDC-CC thus forms generation 1 (G1) in MOS.

### Recruitment: offspring generations 2 and 3

Children (G2) and grandchildren (G3) to index individuals in G1 are recruited, using official register information from the Swedish Tax Agency. At the start of the study in 2013, 10,202 individuals were identified as potential participants. Invitations are sent by mail and followed up by phone calls following full ethical approval of the study. All individuals were given written and oral information before signing a detailed informed consent, also covering biobank storing. The informed consent included, for instance, information about biobank storage and genetic analysis. The first 150 participants were recruited based on that their first-generation index-individual (G1) had an early cardiac event (myocardial infarction or stroke, men < 60 years, women < 65 years). These individuals were specially recruited to enable studies on metabolic difference between individuals with a positive family history of cardiovascular events and those without*.* G2 and G3 individuals are recruited if 18 years or older and living in Malmö or the nearby catchment area. The geographical inclusion criteria were expanded in April 2017 to the region of Skåne, southern Sweden. No exclusion criteria are applied except difficulties in understanding information in Swedish.

### Ethical considerations

We obtained ethical approval for MOS from the Regional Ethics committee (REPN) in Lund (Dnr. 2012/594), as linked to the obtained ethical approval for re-examination of the parents (G1) in the MDC Cardiovascular arm cohort 2007–2012 (Dnr. 532/2006) and the original application for the MDC baseline examination (LU-51-90). We have also registered the MOS biobank at the Regional and National Biobank Register, Sweden. In addition, the dental sub-study (MODS) has been approved by the Regional Committee for Radiation Protection at the Skane University Hospital (7th November 2013). Early detection of individuals at high cardio metabolic risk from risk families is well appreciated by the participants and ethically justified according to our view.

## Methods

### Clinical assessment

*1a. Anthropometrics* Participants are examined with measurements of height (cm) with their legs together looking straight ahead, in indoor clothing without shoes and hats. Weight (kg) is measured on a calibrated balance beam or digital scale. To measure hip circumference (cm), the individuals stand erect with arms at the sides, feet together, when the maximum circumference over the buttocks are measured. Waist (cm) is measured midway between the lowest rib margin and the iliac crest. Resting blood pressure (mmHg) and heart rate (beats/min) is measured as a mean of two readings in the supine position after 10 min rest by use of an automatic device (Omron).

*1b. Blood samples* These are drawn during the first day visit. In total, nine test tubes are filled with a total of 50 ml. Participants are instructed to be fasting since 10 p.m. the previous day but allowed to drink water. Fasting blood samples are analysed for lipids, glucose and creatinine at the Department of Clinical Chemistry, Malmö. In addition, aliquots of blood, serum and plasma, as well as buffy coat from EDTA-blood for DNA extraction, are stored in − 80 °C in a local biobank (BD47), run by the Region Skåne County Council. Later on, the protocol was changed so that also whole blood samples are sent to the Department of Clinical Chemistry and further stored in the biobank, details about the analysis performed the Department of Clinical Chemistry are described in Supplementary Table S1. We aim to have full genome-wide (GWAS) genetic data on all MOS participants, as is already the case for the first generation (GI) in the MDCS.

*1c. Cardiovascular and pulmonary phenotyping *For arterial characterization, seven different methods are used (listed in Table 1). These include ultrasound of the *arteria carotis* (Logiq E9, GE Healthcare), and the assessment of arterial stiffness with pulse wave velocity (PWV), as well as pulse wave analysis (PWA), by use of Sphygmocor^®^ (AtCor, Australia). In addition, we evaluate peripheral finger blood flow by EndoPat^®^ (Itamar, Israel). The ankle-brachial index is measured by Doppler (Hadeco Bidop ES 100V3). In G2, 24-h ambulatory blood pressure is measured and by indirect methods, also central blood pressure and arterial stiffness are calculated (24-h Arteriograph^®^). Cardiac size and function is assessed by performing *echocardiography* (Vivid 7, GE Healthcare) and 24-h Holter ECG in a sub-sample of both G2 and G3.

**Table 1:** Cardiovascular, hemodynamic and metabolic phenotyping in MOS (for details see “Appendix”)

Method	Device	Examined
Ultrasound of the carotid arteries	Logiq E9 (GE Healthcare)	G2
Pulse wave velocity (PWV)	Sphygmocor (AtCor, Australia)	G2 & G3
Pulse wave analysis (PWA)	Sphygmocor (AtCor, Australia)	G2 & G3
Ankle Brachial Pressure Index (ABPI)	Sphygmomanometer and pen Doppler Hadeco Bidop ES-100V3	G2 & G3
Endothelial function	EndoPat (Itamar, Israel)	G2
Skin Auto fluorescence of Advanced Glycation End (AGE) products	AGE Reader (DiagnOptics, The Netherlands)	G2 & G3
Ambulatory blood pressure and arterial stiffness	TensioMed Arteriograph 24 (TensioMed Ltd, Hungary)	G2
Echocardiography	GE Vingmed Vivid 7 Ultrasound (GE, Vingmed Ultrasound, Horten, Norway)	G2

^*^G2, generation 2; G3, generation 3

*1d. Pulmonary function* Pulmonary function is tested using screening spirometry (Jaeger Masterscope).

*1e. Skin autofluorescence* Advanced Glycation End (AGE) products is measured by the AGE Reader^®^ (DiagnOptics Technologies, Groningen, Netherlands).

For detailed information about each technical method, see “Appendix 1”.

## Lifestyle

Lifestyle is assessed by an extensive web-based questionnaire, the included sub-domains are listed in Table 2.

**Table 2:** Lifestyle questionnaire sub-domains

Parts included in the questionnaire	
Family relatedness	Women’s reproductive health
Education and professional life	Tobacco
Medical history	Alcohol
Family disease history	Self-perceived health, stress and social life
Irritable bowel syndrome (IBS) VAS*-scale	Physical activity
Medication	Sleep habits

*Visual Analogue Scale (VAS)

### Cognitive testing

Global cognitive function is assessed with the *Montreal Cognitive Assessment (MoCA)* instrument, translated into Swedish. The MoCA test covers various cognitive domains such as memory, visuospatial ability, executive function, language, and attention [18]. The score ranges from 0 to 30 points, where higher scores denote better cognitive function. The test is widely used and has been demonstrated to detect mild cognitive deficits in older adults with high sensitivity [19]. The MoCA test is performed in G2 only.

In addition, attention is measured by the *Symbol Digit Modalities Test (SDMT),* which consists of tasks of simple substitution where a reference key is given to pair geometric figures with numbers during 90 s. Further, cognitive speed (executive function) is measured by *A Quick Test (AQT),* a validated and sensitive test for detection of cognitive impairment independent of education and gender [20]. The result is measured in seconds, and a shorter time needed to full-fill the task indicates a better performance [21]. These two tests are carried out in both G2 and G3,

### Faeces, saliva and urine collection

*Faecal samples:* Collection is done by the participants at home, dispersed in four aliquots in plastic tubes (54 × 28 mm, Sarstedt AB, Sweden) and stored in a freezer (− 20 °C) before brought to the clinic and frozen at − 80 °C. Instructions on how to collect the samples are provided via an instruction video during the first visit at the clinic. The aliquots are finally stored in a central biobank at − 80 °C.

*Saliva samples:* Saliva is collected at the clinic since October 2014, where the participants are given a chewing gum to stimulate saliva production. In total, 10 ml saliva is collected and stored at − 80 °C.

*Urine samples:* Samples are collected by the participants as over-night urine at home the night before the next (second) visit to the clinic. Research staff measure the volume that is aliquoted into five test tubes à 200 ml, which are then stored at − 80 °C in the central biobank.

### Dietary assessment

To assess dietary intakes and habits, a combination of two methods is used. Firstly, a web-based 4-day food record designed by the Swedish National Food Agency (called *“Riksmaten 2010”* in Swedish) is used to capture absolute dietary intake during a four-day observation period. Before the food recording, the participants are tutored via an instruction video (https://www.youtube.com/watch?v=DB3bzD0FJMg) to register everything they eat and drink during four consecutive days, starting the day after their first visit to the clinic, in order to get a representation of all weekdays in the cohort. To help the participants to register as correctly as possible, they are provided with a notebook and a photo book with portion sizes, identical to what are found on the online registration page.

Total energy intake as estimated from the “Riksmaten2010” has been validated with the double labelled water technology (r = 0.40) [22]. Secondly, a Short Food Frequency Questionnaire (SFFQ) is filled in to capture habitual intake of irregularly eaten food items that may not be covered during the 4-day food record period, such as fish. The FPQ covers 32 questions about foods, 3 about beverages, 4 about meal type and 4 about food-related products and a final question about diet-change. The participants are asked to estimate their habitual intake for the previous six months.

At a re-examination sub-study, 400 participants were invited to record their diet once again approximately two years after their initial baseline registration.

### The dental sub-study (MODS)

*The Malmö Offspring Dental Study (MODS)* is the dental arm of MOS initiated in October 2014. MODS participants are recruited from the individuals already enrolled in MOS.

*Oral biofilm samples:* Collection of biofilm in the buccal mucosa and on the tongue dorsum is performed using a Catch-All Sample Collection Swab. After swabbing, the sponge is put back in its vehicle and frozen at − 80 °C. The mesiolingual surface of teeth 16, 26, 36 and 46 is scraped with a sterile Gracey curette to remove subgingival plaque followed by immersing the tip of the curette into 150 μl of lysis buffer [23] on ice after which the tubes are frozen at − 80 °C. If an index tooth is missing, the tooth mesial to the index tooth is chosen. Saliva was sampled by allowing the participant to chew on piece of sterile paraffin tablet for 5 min. The saliva was aliquoted and frozen at − 80 °C for further analysis, including microbiome sequencing.

*Radiologic and clinical examination:* One panoramic radiograph is taken to get an overview of all teeth and alveolar bone. Four bite-wing radiograph pictures of the molar and pre-molars are taken to assess primary caries and alveolar bone height in more detail. Clinically, caries is detected using standard clinical criteria aided by mirror, probe (Hu-Friedy EXD57) and bite-wing radiographs. Cavitated lesions that extend into the dentin are recorded as manifest caries. Periodontal disease is assessed using a periodontal probe with 1 mm grading (Hu-Friedy PCPUNC157) and pockets > 2 mm is recorded at six sites per tooth as well as clinical attachment loss, bleeding on probing and presence of plaque based on the Silness-Löe index. Periodontitis is diagnosed and classified to moderate or severe based on the American Association of Periodontology definitions [24]. Salivary secretion rate is measured through stimulated salivary secretion.

*Dental register data* The Swedish Dental Register (Tandhälsoregistret) contains data on number of teeth and treatments performed due to e.g. periodontal disease or caries.

### Statistics and power calculation

To fully utilize the information collected in our cohort, and to accommodate covariate effects, heritability will be estimated using a variance component model [25]. This model can accommodate pedigrees of any configuration and is well suited for the analysis of extended pedigrees. Relationships between the phenotypes in G2–G3 and disease history in G1 will be evaluated using multivariable logistic regression. We have based our sample size of the G2–G3 on the following rough assumptions (being very well aware that the exact figures vary between phenotypes/diseases): (1) a typical heritability of chronic diseases and their major risk factors is commonly around 50% (20–80%), and (2) a typical point effect estimate (odds ratio or hazard ratio) of one standard deviation change of a biomarker (e.g., plasma biomarker or composite gene score) in relation to risk of future development of a disease is 1.25. Making specific power calculations regarding all the various phenotypes in relation to their co-variation with intergenerational clustering of disease expression is unreasonable.

Given the assumptions stated above, a logistic regression of a binary response variable (Y) on a continuous, normally distributed variable (X) with a sample size of 4000 observations achieves 92% power at a 0.05 significance level to detect a change in Prob(Y = 1) from the value of 0.080 at the mean of X to 0.098 when X is increased to one standard deviation above the mean. This change corresponds to an odds ratio of 1.25. An adjustment was made since a multiple regression of the independent variable of interest on the other independent variables in the logistic regression is assumed to obtain an R-Squared of 0.20.

As this is a family-based study the variation within the cohort could be expected to be lower than in a general population, why this should be taken into account. One way to deal with this bias is to use a robust standard error based on the family structure in the cohort.

## Results

### Initiating phase

The first study year (2013–2014) was an initiating phase of MOS and after this period the main study protocol was set, with the main changes including digitalization of questionnaires, removal of the oral glucose tolerance test, and a change in blood sample preparation.

### Interim analysis

In May 2017, the MOS study had reach halftime according to initial recruitment goals, with a participation rate of 47.0% and an age span of 18–71 years. Of the 2644 participants, 1326 were G2 and 1321 were G3. The 2644 participants originate from 1387 families of which 56% include at least two, and 28% at least three individuals, from the same family or extended family, the largest five families having nine recruited participants each. The participants have provided a number of different biological samples and other data, Table 3.

**Table 3:** Number of individuals (n, percent) included in the different sample and data collections at interim analysis (November 2017)

Faecal samples	2351 (89%)
Urinary samples	2459 (93%)
Dietary records	1791 (68%)
Diet questionnaire	2072 (78%)
Lifestyle questionnaire—basic version	2242 (85%)
Lifestyle questionnaire—extensive version	2137 (81%)

This first interim analysis of MOS identifies a cohort characterized by a wide age range (18–71 years), (52% women), slight overweight (mean BMI 25.8 kg/m^2^), a dominance of non-smokers (60% have never smoked), 38% have a university degree, and 28% are doing some exercise every week. Further descriptive background data on the study population is presented in Table 4.

**Table 4:** Characteristics of MOS participants (n = 2644) at interim analysis

	Mean (SD) or %	Generation 2	Generation 3	Men	Women
Age (years)	39.8 (13.9)	51.5 (8.1)	27.9 (6.7)		
Sex (% women)	52.1	52.3	52.1		
BMI (kg/m^2^)	25.8 (4.7)	26.8 (4.7)	24.9 (4.6)	26.6 (4.4)	25.1(4.9)
Waist circumference (m)	0.85 (0.09)	0.93 (0.13)	0.86 (0.12)	0.95 (12.8)	0.84 (12.42)
Fasting glucose (mmol/L)	5.48 (1.05)	5.7 (1.28)	5.3 (0.73)	5.57 (1.2)	5.4 (0.9)
LDL cholesterol (mmol/L)	3.13 (0.94)	3.42 (0.95)	2.83 (0.84)	3.27 (0.99)	3.0 (0.9)
HDL cholesterol (mmol/L)	1.61 (0.48)	1.66 (0.52)	1.57 (0.44)	1.41 (0.39)	1.80 (0.48)
Triglycerides (mmol/L)	1.12 (0.69)	1.28 (1.57)	1.01 (0.58)	1.32 (1.32)	0.99 (0.53)
Systolic Blood Pressure (mmHg)	115 (15)	121 (16)	111 (11)	121 (12)	112 (15)
Diastolic Blood Pressure (mmHg)	71 (10)	76 (10)	67 (7)	72 (10)	71 (10)
Medical history					
Any prescribed drug (%)	37.1	45.8	27.4	24.3	35.9
Antibiotics use the last 6 months (%)	14.2	11.7	17	11.1	16.9
Prevalent diabetes (n)	85	69	16	53	32
Prevalent cancer (n)*	192	149	45	31	161
Prevalent CVD events (n)**	38	34	4	22	16
Lifestyle, education					
Physical activity at work (%)					
Very light	44	48.4	39	45.7	42.1
Light	15.2	17.2	12.8	13.3	17
Moderate	24.7	22.5	27.2	19.6	29
Heavy	12.9	9.8	26.4	14.7	11.3
Very heavy	3.3	2.1	4.6	6.7	0.6
Leisure physical activity (%)					
Sedentary	9	9.5	8.4	10.1	8.1
Low	37.5	40.2	34.4	34.9	40.1
Moderate	27.9	28.1	27.7	27	28.5
High	25.6	22.3	29.5	28	23
Smoking (%)					
Regular smokers	7	6.9	7	5.5	8.2
Irregular smokers	9.8	5.8	14	10.7	9
Ex-Smokers	24.2	33.4	14.6	33.4	25.8
Never smokers	59	53.9	64.4	61.4	57
Snuff use (%)					
Ever used daily snuff	19.9	21.6	18	36	6.1
Alcohol intake (%)					
Never	7.1	6.7	7.6	5.7	8.4
1/month	22	17.6	27.4	16	27.1
2–4/month	41.3	36.1	47	43.9	39.3
2–3/week	25.7	34.8	16.3	29.1	22.8
> 4/week	3.8	5.8	1.6	5.3	2.4
Education level (%)					
Basic < 9 years	0.2	0.18	0.3	0.19	0.33
Primary school 9–12 years	6.8	8.7	4.6	7.1	6.7
High school	55.1	50.5	60.2	62.3	50.1
University	37.9	40.6	34.9	30.5	42.8

Means (SD) and proportions (%)

LDL: low density lipoprotein; HDL: high density lipoprotein

*prevalent tumours at baseline

**CVD event = cardiovascular event (myocardial infarction and/or stroke)

### Present status of MOS

As of May 2020 we have recruited in total 4721 participants (G2: 2609; G3: 2112). The participation rate is 46.8% (G2: 54.3%; G3: 40.0%). Clinical and technical data have to be assessed for quality control as similar to the interim analysis in November 2017.

### Dietary data

**Fig. 1:**
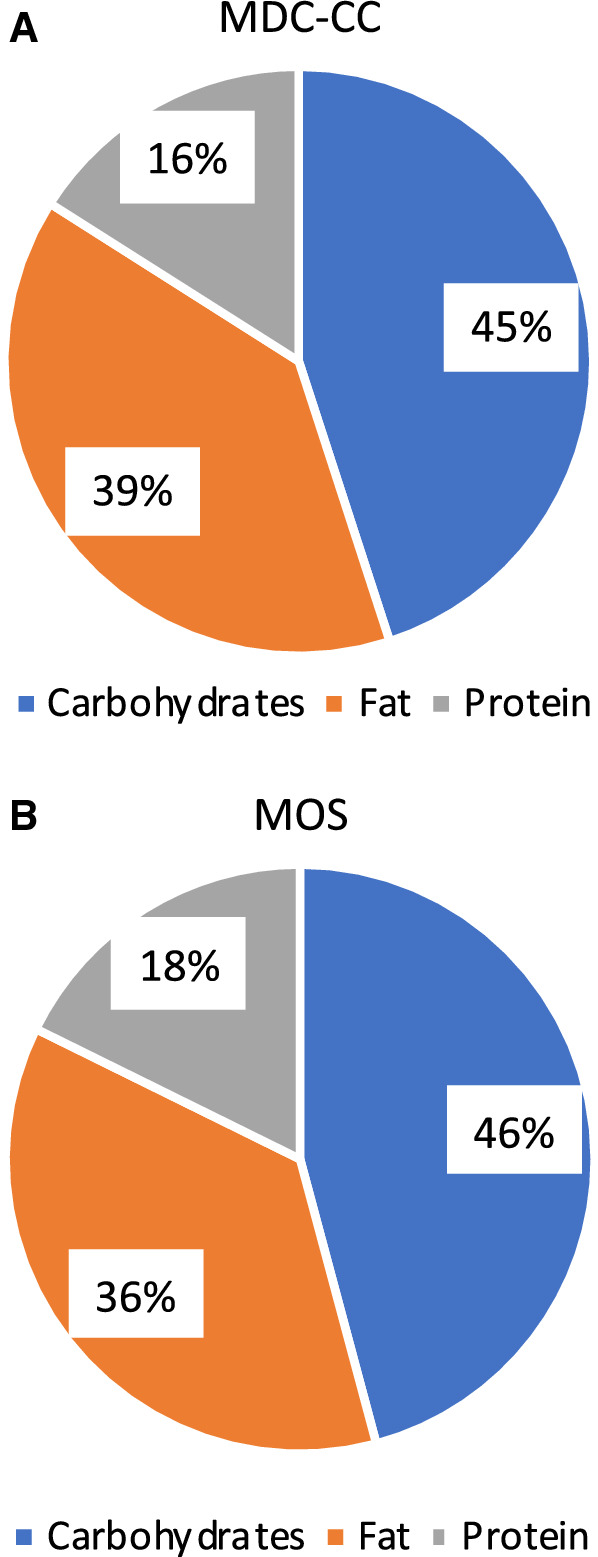
Macronutrient intake in generation 2/3 (MOS) and generation 1 (MDC-CC)

In these interim analyses, diet was very similar between the generation 1 MDC-CC and generation G2 and G3 in MOS, both for macronutrient intake and the estimated daily intake of energy (kcal) and dietary fibres per day. In MDC-CC these figures were 2341 kcal and 22 g, and in MOS they are 2050 kcal and 18 g, respectively (Fig. 1).

### Vascular data

In two publications the arterial stiffness (carotid-femoral pulse wave velocity, c-f PWV) and related measures (Augmentation Index, Aix) in MOS have been used. In the first paper Sperling et al. [26] could show that early life factors such as birth weight adjusted for gestational age were predictive of adult life c-f PWV and Aix, and in the second paper Petersson Rosberg et al.[27] could show similarities in c-f PWV levels within families across three generations.

### Dental and oral data

Dental data in MODS is available from 831 of the 2644 MOS participants based on the halftime data (2017). A low salivary secretion rate of < 0.7 ml/min was observed in 8.7% of the participants and the mean bleeding on probing was 28.9% (SD 18.3) in the MODS cohort. Moderate periodontal disease manifestations were observed in 24.8% and severe periodontal disease in 6.5% of the participants. Manifest caries lesion was diagnosed in 24.8%, defined as lesions clearly involving dentin or fractured or missing filling. The mean number of teeth were 27.2 (SD 1.6) (Supplementary Table S1).

In the additional questionnaire in MODS, 90.6% of participants reported being delivered through vaginal birth and 6.3% following caesarean sectio. In addition, 80.1% reported being breastfed and 6.6% had received formula feeding.

In a recent publication by Jönsson et al., the association between periodontitis and changes of the carotid arterial wall (plaque area) has been shown [28]. 2455 MOS participants (93%) had available dental data in The Swedish Dental Register.

### Gut microbiota

**Fig. 2:**
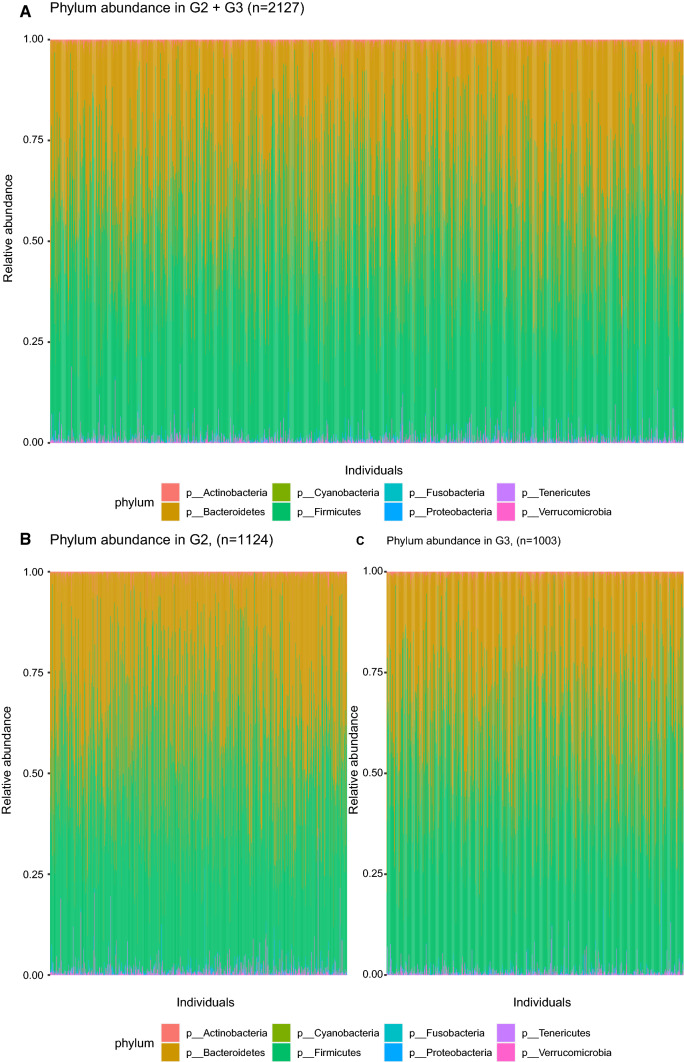
Bacteria at phylum level in **a** MOS (n = 2200), **b** G2, and **c** G3

Of the 2351 faecal samples collected in the MOS halftime cohort, 2200 were sequenced between 2013 and 2017. The microbial DNA was extracted using the QIAamp column Stool Kit. The V1-V3 region (300 bp × 2) of the 16S rRNA gene was amplified and sequenced on a HiSeq Illumina at the GATC Biotech (Constance, Germany) and the sequences were matched with the Greengenes database (version 13.8). In this study sample the average amount of counts per sample were 434,008 with a maximum of 1,268,483, and 533 operational taxonomical units (OTU) were detected with an average of 90 OTUs per individual. With 16S sequencing, we get reliable data of bacteria on 5 taxonomical levels (phylum, class, order, family and genus), and for some bacteria, estimates can be done down to species level. The two dominant phyla are Firmicutes and Bacteroidetes (Fig. 2), and the three most common genera are Bacteroides, unclassified genus in the Ruminococcaceae family and an unclassified genus in the Rikenellaceae family.

In one publication by Ottosson et al.[29] the association between obesity and microbiota patterns were described. The authors discovered associations between four gut microbiota genera (*Blautia, Dorea*, Ruminococcus, and SHA-98) and BMI-predictive plasma metabolites, including glutamate and BCAAs. Thus, these metabolites could be mediators between gut microbiota and obesity, pointing to potential future opportunities for targeting the gut microbiota in prevention of obesity [29]. Additionally, Eriscon et al. investigated the associations between gut bacteria, a data driven healthy eating pattern and prediabetes. In this paper we among other things observed *Roseburia* to be associated both with a decreased risk of prediabetes and with a higher adherence to a healthy diet pattern [30].

### Gastrointestinal aspects

In one paper by Ohlsson et al. [31] the biomarker zonulin, a biomarker of supposed increased intestinal permeability, was investigated in a subgroup of MOS participants. Higher zonulin levels were associated with higher waist circumference, diastolic blood pressure, fasting glucose, and increased risk of metabolic diseases, but not with gastrointestinal symptoms.

### Follow-up data on morbidity and mortality

From the Swedish national registers, we have provided follow-up data for 2644 participants until the 31st of December 2016. During the mean follow-up time of 1.6 (0–3.8) years, 23 incident diabetes diagnoses, 22 incident malignancies, 32 cardiovascular events and 5 deaths were recorded within the study population. Further follow-up data will be available in the near future.

## Discussion

With the ambition to characterize family patterns of chronic cardio metabolic disease conditions in search of the so called “*missing heritability*”, MOS had in 2017 recruited 2644 individuals (47% attendance) representing children (G2) and grandchildren (G3) to individuals (G1) first examined within the MDC-CC study between 1992 and 1996. We collect a wide range of data including somatic phenotyping, lifestyle characteristics and dietary profiling, as well as collection of faecal and oral samples for microbiome analysis. Genetic data (GWAS) will be analysed in all MOS participants, thereby increasing the possibilities to elucidate on gene-environmental interactions.

Some data have already been published concerning the role of the biomarker zonulin in relation to features of the metabolic syndrome and gastrointestinal symptoms [31], and the link between BMI-related blood metabolite pattern and gut microbiota composition [29]. In addition, we have published data on the self-reported family history of cardio metabolic disease in the index generation (G1) from MDC-CC [32], but also on the genetic influence of hyperglycaemia on arterial stiffness based on a genetic risk score in the same G1 index subjects [33]. Newer studies have documented family patterns of arterial stiffness [26] and the association between periodontitis and carotid plaque area [28].

In the 2200 sequenced faecal samples, the two dominant phyla were as expected *Bacteroidetes* and *Firmicutes*, and the relative abundance of different genera were comparable to what others have identified by 16S sequencing in European population [34]. In our study, the V1–V3 region was sequenced to get long reads and OTUs were picked with a “closed method” using the Greengene database. Today, the more comprehensive method of shot-gun metagenomic sequencing has drastically decreased in price and will be the first hand option for sequencing of future samples. However, the 16S methods is still very relevant as it provides reliable data down to genus level, takes up much less data capacity and many other larger studies have 16S data which enables comparison and generalization.

Similar studies exist, both the Framingham Offspring Study (FOS) in the US [12, 13] and the LifeLines study in the Netherlands [34, 35], both focusing on family traits of cardiovascular risk, and for LifeLines also on gut microbiota [34]. MOS is similar, yet different form these studies, with the unique possibility of national register-based follow-up. This allows tracing of the incident fatal and non-fatal events by local, regional and national registers, and not only by medical records of hospitalization that provide an underestimate of the true incidence rate of for example T2D, which is often diagnosed and treated in primary health care only. In addition, the source population (G1) is very deeply phenotyped and genotyped. The long follow-up has provided valuable information on G1 morbidity and mortality, and an extensive dietary data was assessed at baseline. Finally, we have recently got access to genome-wide genotyping and exome sequencing data in G1 (O Melander, personal communication), which provides information on parental and grandparental genetics and allows genotype-based recalling of MOS participants. However, there are also many similarities between these three population-based family studies making comparisons between them possible. In addition, also the Reykjavik Heart Study (RHS) includes some information on family traits, possible to explore and compare with, as was already done in the past when comparing echocardiography findings in the RHS with MPP [36].

### Strengths and limitations of MOS

The *strengths* of MOS include the deep phenotyping over three generations in combination with extensive data on medical history, food habits, lifestyle and genetics, but also on gut and oral microbiota in children and grandchildren (G2, G3). These data can help us to elucidate on the so-called missing heritability when we are going to study the family patterning of these characteristics. One *limitation* of MOS is the less than optimal participation rate (47% in general, but 54% in G2 and 40% in G3) that reflects the difficulties in constructing attractive-enough invitations directed to young or middle-aged subjects in a changing urban society, as many of the younger subjects feel healthy. Other limitations are that not all subjects provide neither all dietary data nor faeces samples, and that we were not able to offer all clinical investigations to both children (G2) and grandchildren (G3) due to cost constraints.

Future research in MOS will enable us to investigate novel aspects of the genetics-diet-microbiota patterns and how these connect to both vascular and metabolic traits, for example arterial stiffness as a marker of vascular ageing. Importantly, these patterns will be prospectively associated to incident disease and mortality endpoints derived from national registers. Moreover, as we have been able to trace early life data such as birth weight and length in almost all study participants from the Medical Birth Register, connecting these data to organ function, as well as morbidity and mortality outcomes, can bring new understanding regarding the early life developmental origin of adult health and disease [37]. One such study based on MOS was recently published [26]. Finally, many studies have associated obesity, T2D and CVD with specific changes of the gut microbiota composition and novel mechanisms have emerged in particular from preclinical gut microbiota studies. MOS will offer what the field is still missing, a large population- and family-based prospective study, with multiple omics data and clinical phenotyping, where causal associations and directions can be investigated utilizing the Mendelian randomization approach.

In summary, MOS is a large ongoing population-based, transgenerational cohort study generating a rich set of data, including extensive phenotyping for vascular function, metabolic traits, genotyping as well as faecal metagenomics and dietary assessment, and has at half time included 2644 individuals. This cohort, aiming to have up to 6000 participants by 2021, will enable an extensive number of studies with focus on investigating heritability and prediction of multifactorial diseases such as T2D and CVD.

### Electronic supplementary material

Below is the link to the electronic supplementary material.

Supplementary material 1 (DOCX 18 kb)

## Appendix 1: Description of technical methods

### Ultrasound of the carotid arteries

The degree of atherosclerosis is measured in the carotid arteries using B-mode ultrasound (Logiq E9, GE Healthcare). Images of the left and right common carotid artery and bifurcation are captured in end-diastole. Intima media thickness (IMT) is subsequently measured off-line using a semi-automatic analysis program (Artery Measurement System [AMS]), as a sign of early stages of atherosclerosis. A plaque is defined as a focal thickening of the intima layer exceeding 1.2 mm in height. All plaques present in the visible parts of the common, internal and external arteries and in the bifurcation are measured online (height, length and area) as a sign of more advanced atherosclerosis. Morphology of the plaque is measured on-line in terms of ulceration and assessment of the plaque surface being smooth or uneven. Further measurements of plaque morphology will be done off-line using the AMS system. Blood flow velocity in the internal carotid artery is measured for evaluation of clinical significant degree of stenosis. Direction of the blood flow velocity is in the vertebral artery is documented.

### Pulse wave velocity

Arterial stiffness is assessed using the Sphygmocor (AtCor, Australia) device [27]. Pulse wave velocity (PWV) in m/sec, is measured along the abdominal aorta. Pulse curves are obtained from the carotid and the femoral arteries. The device calculates the time from the R-wave, on a simultaneous ECG-recording, to the foot of the pulse wave of the carotid and femoral arteries, respectively. The distance, measured as the straight line from the carotid to the femoral artery times 0.8, is entered manually in the program, as recommended in guidelines. Two measurements are performed. If the difference between the two measurements is above 0.5 m/s a third is performed. The mean value of the measurements is used.

### Pulse wave analysis

Pulse wave analysis (PWA) is performed using the pulse curve of the radial artery obtained by the SphygmoCor transducer. Two measurements are performed. If the difference between the two measurements is above 3% a third is performed. The mean value of the measurement is used.

### Ankle Brachial Pressure Index

A measurement of the Ankle Brachial Pressure Index (ABPI) is performed and measured using cuffs of an appropriate size attached to a sphygmomanometer. Systolic blood pressure is measured using a pen Doppler (Hadeco Bidop ES 100V3). Blood pressure cuffs are attached to the upper arms and the ankles. Systolic blood pressure is measured beginning in the right arm, then in the right ankle, the left ankle and finally in the left arm. Pressure is measured once in each arm over the radial artery. In the ankle the pressure is measured once in *art. dorsalis pedis* and then once in *art. tibialis posterior*. ABPI is calculated by dividing the highest blood pressure on each ankle with the blood pressure from the arm with the highest value.

### Endothelial function

Endothelial function is measured using the EndoPat device (Itamar, Israel) to estimate the endothelium-dependent vasodilation following post-ischemic hyperaemia. A cuff is placed on the non-dominant upper arm, and the index or middle fingers are placed in pneumo-electric tubes. Arterial pulsatile volume changes are recorded continuously. After a 5 min period of rest, the cuff is inflated to 200 mmHg, with the opportunity to increase the pressure to a maximum of 300 mmHg if necessary. After occlusion for 5 min, the pressure of the cuff is released and the arterial dilatation mediated by the occlusion, assessed as an increase in the signal amplitude, is recorded for another 5 min. The reactive hyperaemia index (RHI) is calculated as a post-occlusion to pre-occlusion ratio of the signal amplitudes.

### Ambulatory blood pressure and arterial stiffness measurements

Ambulatory blood pressure and arterial stiffness are measured in subgroups using the TensioMed Arteriograph 24 (TensioMed^™^ Ltd, Hungary) device during 24 h. Blood pressure is then measured every 15 min during daytime (06.00–22.00) and every 30 min during night (22.00–06.00). Brachial systolic and diastolic blood pressure, aortic systolic and diastolic blood pressure, aortic PWV and heart rate are recorded at each measurement. Subjects are excluded from analyses if more than 30% of the blood pressure recordings were missing. Both blood pressure and indirectly calculated aortic PWV by use of Arteriograph 24 have been validated against standardized methods previously.

### Echocardiography

Conventional echocardiograms were carried out using a GE Vingmed Vivid 7 Ultrasound (GE, Vingmed Ultrasound, Horten, Norway) with a 1–4 MHz transducer (M3S). Cine loops were obtained from standard views (parasternal long axis, apical 4- and 2-chamber). Measurements of chamber size and function were done offline using Philip Intellispace Cardiovascular (Philips Medical Systems, Netherlands) according to the recommendations of the American Society of Echocardiography [38]. Internal left and right ventricular dimensions were measured from parasternal long axis view at end-diastole. Left ventricular volumes were calculated using the biplane Simpson method of disks, by manual tracing in two-dimensional end-diastolic and end-systolic frames defined as the largest and smallest left ventricular cavities, respectively, in apical 4- and 2-chamber projections. Ejection fraction (EF) was calculated automatically from end-diastolic volumes (EDV) and end-systolic volume (ESV) using the following formula: EF = (EDV- ESV)/EDV.

### Spirometry

A pulmonary function test is performed using spirometry (Jaeger Masterscope) where forced vital capacity (FVC) and forced expiratory volume in 1 s (FEV_1_) are measured. Three acceptable measurements are required where the difference between the highest and second highest value should be ≤ 150 mL (or ≤ 100 mL if FVC ≤ 1L).

### Skin autofluorescence

Skin autofluorescence of Advanced Glycation End (sf-AGE) products is measured using an AGE Reader (DiagnOptics, Groningen, The Netherlands) at room temperature. During measurement, a skin surface of approximately 2 cm^2^ is illuminated with an ultraviolet light source that ranges between 300 to 420 nm, when the skin of subjects emits light at different wavelengths that are registered by a spectrometer. The major contribution in s-f AGE comes from AGEs linked to collagen and is influenced by glycaemia and oxidate stress, but also dietary components. An average of three readings on subject’s non-dominant forearm is calculated and reported in arbitrary units (AU). Measurements of the s-f AGE accumulation using the AGE-Reader have previously been validated.
