# Supplementary material for: The Malmö Offspring Study (MOS): design, methods and first results
Source: Eur J Epidemiol. 2020 Nov 21;36(1):103–16. doi: 10.1007/s10654-020-00695-4 (PMC7847466; doi:10.1007/s10654-020-00695-4)
Supplement: Supplementary file 1 — Supplementary material 1 (DOCX 18 kb) [file 10654_2020_695_MOESM1_ESM.docx]

**Supplementary Table S1**

**Characteristics of oral and dental health in individuals of the Malmo Offspring Dental Study (MODS) with means (SD) and proportions.**

|  | **Means (SD), %** |
| --- | --- |
| N | 831 |
|  |  |
| Bleeding on probing mean, (SD) | 28.9 (18.3) |
| No periodontitis, n (%) | 571 ( 68.7) |
| Moderate periodontitis, n (%) | 206 (24.8) |
| Severe periodontitis, n (%) | 54 (6.5) |
| Hyposalivation, n (%) | 72 (8.7) |
| Manifest caries /tooth fracture, n (%) | 206 (24.8) |
| Number of teeth (excluding wisdom teeth), mean (SD) | 27.2 (1.6) |
